# Alignment of stroma fibers, microvessel density and immune cell populations determine overall survival in pancreatic cancer—An analysis of stromal morphology

**DOI:** 10.1371/journal.pone.0234568

**Published:** 2020-07-13

**Authors:** Louisa Bolm, Petro Zghurskyi, Hryhoriy Lapshyn, Ekaterina Petrova, Sergiy Zemskov, Yogesh K. Vashist, Steffen Deichmann, Kim C. Honselmann, Peter Bronsert, Tobias Keck, Ulrich F. Wellner

**Affiliations:** 1 Department of Surgery, University Medical Center Luebeck, Luebeck, Germany; 2 Department of General Surgery #1, Bogomolets National Medical University, Kyiv, Ukraine; 3 Department of Pathology, Medical Center - University of Freiburg, Faculty of Medicine, University of Freiburg, Freiburg, Germany; 4 Tumorbank Comprehensive Cancer Center Freiburg, Medical Center - University of Freiburg, Faculty of Medicine, University of Freiburg, Freiburg, Germany; Centro Nacional de Investigaciones Oncologicas, SPAIN

## Abstract

**Introduction:**

The aim of this study was to define histo-morphological stroma characteristics by analyzing stromal components, and to evaluate their impact on local and systemic tumor spread and overall survival in pancreatic ductal adenocarcinoma (PDAC).

**Methods and materials:**

Patients who underwent oncologic resections with curative intent for PDAC were identified from a prospectively maintained database. Histological specimens were re-evaluated for morphological stroma features as stromal fibers, fibroblast morphology, stroma matrix density, microvessel density and distribution of immune cell populations.

**Results:**

A total of 108 patients were identified undergoing curative resection for PDAC in the period from 2011–2016. 33 (30.6%) patients showed parallel alignment of stroma fibers while 75 (69.4%) had randomly oriented stroma fibers. As compared to parallel alignment, random orientation of stroma fibers was associated with larger tumor size (median 3.62 cm vs. median 2.87cm, p = 0.037), nodal positive disease (76.0% vs. 54.5%, p = 0.040), higher margin positive resection rates (41.9% vs. 15.2%, p = 0.008) and a trend for higher rates of T3/4 tumors (33.3% vs. 15.2%, p = 0.064). In univariate analysis, patients with parallel alignment of stroma fibers had improved overall survival rates as compared to patients with random orientation of stroma fibers (42 months vs. 22 months, p = 0.046). The combination of random orientation of stroma fibers and low microvessel density was associated with impaired overall survival rates (16 months vs. 36 months, p = 0.019). A high CD4/CD3 ratio (16 months vs. 33 months, p = 0.040) and high stromal density of CD163 positive cells were associated with reduced overall survival (27 months vs. 34 months, p = 0.039). In multivariable analysis, the combination of random orientation of stroma fibers and low microvessel density (HR 1.592, 95%CI 1.098–2.733, p = 0.029), high CD4/CD3 ratio (HR 2.044, 95%CI 1.203–3.508, p = 0.028) and high density of CD163 positive cells (HR 1.596, 95%CI 1.367–1.968, p = 0.036) remained independent prognostic factors.

**Conclusion:**

Alignment of stroma fibers and microvessel density are simple histomorphological features serving as surrogate markers of local tumor progression dissemination and surgical resectability and determine prognosis in PDAC patients. High CD4/CD3 ratio and CD163 positive cell counts determine poor prognosis.

## Introduction

Pancreatic ductal adenocarcinoma (PDAC) is one of the most aggressive solid malignancies [1]. The majority of patients present with locally advanced disease or distant metastases at time of diagnosis [2]. Complete oncologic resection is the only curative option in PDAC [3].

Extensive desmoplasia is a characteristic macro- and microscopical PDAC feature [4,5]. PDAC are often composed of high amounts of extracellular matrix and fibroblasts and low amounts of tumor cells and microvessels. Fibroblasts and tumor cells form a complex interaction network and orchestrate the remodeling of the tumor microenvironment by paracrine signaling and matrix remodeling [6,7]. The interaction of local fibroblasts called pancreatic stellate cells, extracellular matrix components and tumor cells in PDAC has been increasingly studied in the past years [6,8,9]. Dense desmoplasia in PDAC is associated with tumor hypoxia and impairs drug delivery [10]. Furthermore, signaling between fibroblasts and PDAC tumor cells is associated with local tumor growth and tumor cell invasion [11,12]. However, complete depletion of PDAC stroma results in more aggressive and invasive tumors in xenograft models [10, 11]. In addition to fibroblasts and stromal elements, immune cell populations such as T cell subtypes and tumor-associated macrophages are present in the stromal microenvironment and contribute to the tumor-stromal-crosstalk [13,14]. PDAC stroma may be regarded as a complex and controversial key player in PDAC progression and the interplay of stroma and tumor cells has not been entirely understood yet.

The main part of data evaluating interaction between PDAC cells, fibroblasts and extracellular matrix is derived from *in vitro* and xenograft models [5,7]. Only few studies assess the impact of desmoplasia in clinical and histopathological settings [15,16]. Additionally, the pathological work-up of stroma features has not been introduced to clinical routine yet. Currently, no systematic scoring system for distinct aspects of PDAC desmoplasia is available.

The aim of this study was to perform a morphological analysis of PDAC stroma analyzing stromal matrix, vasculature and stromal cell populations. Furthermore, we intended to define simple histopathological stroma assessment criteria and aim to evaluate the impact of stroma features on patient prognosis.

## Methods and patients

### Patients and study parameters

**Fig 1:**
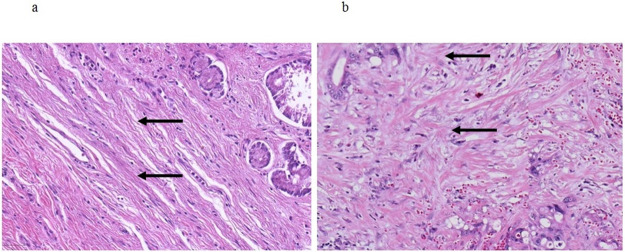
Alignment of stroma fibers. a Parallel alignment of stroma fibers, arrows pointing at elongated parallel oriented extracellular matrix fibers; b Random orientation of stroma fibers, arrows pointing at extracellular matrix fibers aligned at an angle of more than 45°. 20x magnification.

**Fig 2:**
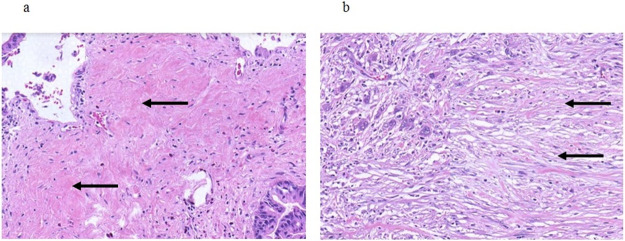
Stromal density. a High stromal density, arrows pointing at dense bunches of extracellular matrix; b Low stromal density, arrows pointing at thin elongated extracellular matrix fibers showing gaps between the single fibers. 20x magnification.

**Fig 3:**
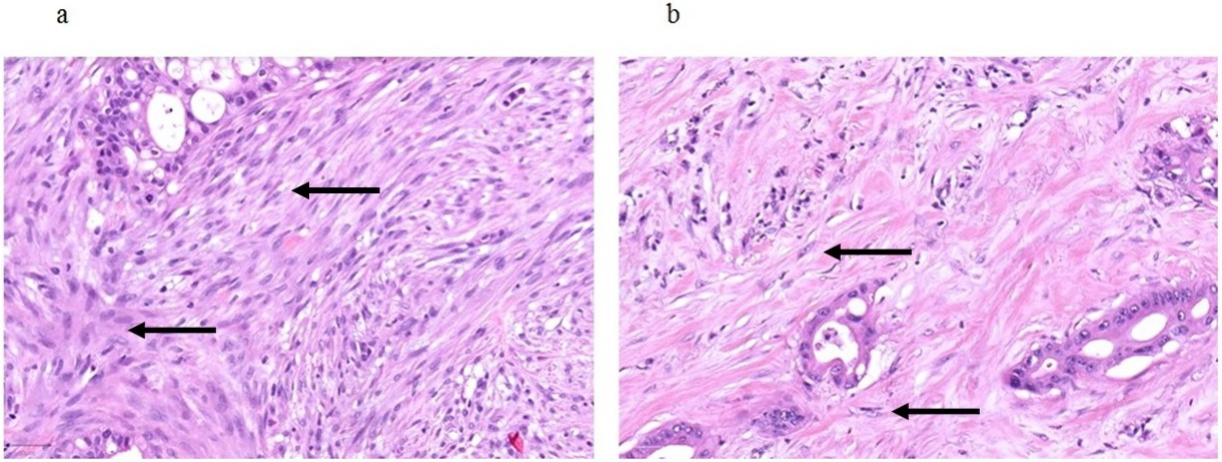
Fibroblast density. a High fibroblast density, arrows pointing at densely arranged fibroblasts; b Low fibroblast density, arrows pointing at rarely scattered fibroblasts throughout the stromal areas. 20x magnification.

**Fig 4:**
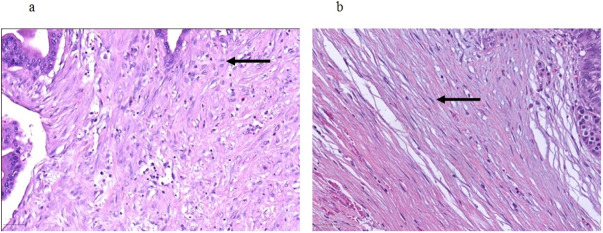
Fibroblast morphology. a Arrow pointing at round-shaped fibroblasts; b Arrow pointing at spindle-like shaped fibroblasts. 20x magnification.

**Fig 5:**
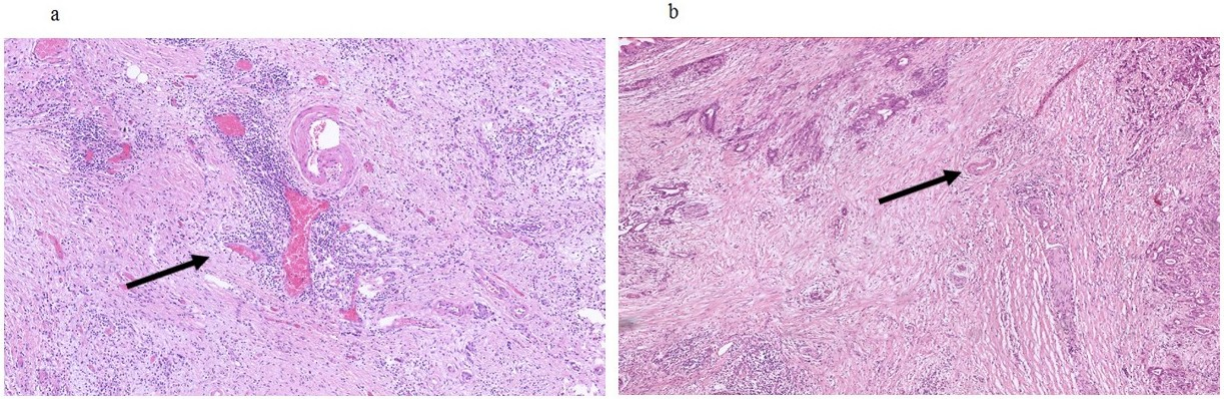
Microvessel density. a High microvessel density, arrow pointing at densely arranged microvessels; b Low microvessel density, arrow pointing at rarely scattered microvessels. 10x magnification.

Patients undergoing oncologic resections with curative intent for PDAC were identified from the prospectively maintained database of the department of surgery at the University Hospital Schleswig-Holstein, Campus Luebeck, Germany, a tertiary hospital and high-volume center of pancreatic surgery. Ethics approval was obtained from Luebeck university ethics committee (#17-118A). Patients in the study were resected in the period from 2011 to 2016. The following patient baseline and surgical parameters were included for the analysis: Gender, age at date of operation and type of resection (Whipple versus pylorus-preserving PD (PPPD) versus left pancreatic resection versus total pancreatectomy (TPE)). Histopathological parameters included in the analysis were tumor size, T stage, N stage, M stage, grading according to Broders [17] and R status. TNM staging was performed according to the 7th edition of the American Joint Committee on Cancer (AJCC) [18]. All histopathological specimens were further examined for stroma morphology by one pathologist and two trained surgeons. Stroma morphology was evaluated in digitalized slides stained for hematoxylin and eosin (H&E). Two representative slides with both PDAC tumor and stroma per patient were re-evaluated for stroma morphology. For processing digitalized samples, the QuPath computer program was used [19]. First, each slide was inspected at 4x magnification, and 5 distinct localizations with pronounced stroma were determined. Each localization was assessed at 20x magnification. Alignment of stroma fibers was assessed as either parallel alignment or random orientation of stroma fibers (Fig 1a and 1b). If most stromal fibers were oriented parallel to each other, alignment was defined as parallel. In case of stroma fibers that were not parallel, but in a more than 45° angle one to another or even chaotic, alignment of stroma fibers was classified as random orientation. In case of heterogeneous stromal alignment within one slide, the slide was classified according to the most frequent stromal orientation. Stromal density was classified as either low or high density (Fig 2a and 2b). The assessment of stromal alignment was repeated after staining for collagen I. Fibroblast density was defined as either low or high (Fig 3a and 3b). Fibroblast morphology was determined as either round or spindle-like (Fig 4a and 4b). For all morphology features the most frequent morphology characteristic in all five localizations was selected. Microvessel density was modified according to Weidner et al. [20]. At 10x magnification, the four stroma localizations with most capillaries and small venules (microvessels) were determined. The number of these microvessels was counted for each localization at 100x magnification and the mean value for all four localizations was calculated. The mean number of microvessel density was dichtotomized according to the median (Fig 5a and 5b).

**Fig 6:**
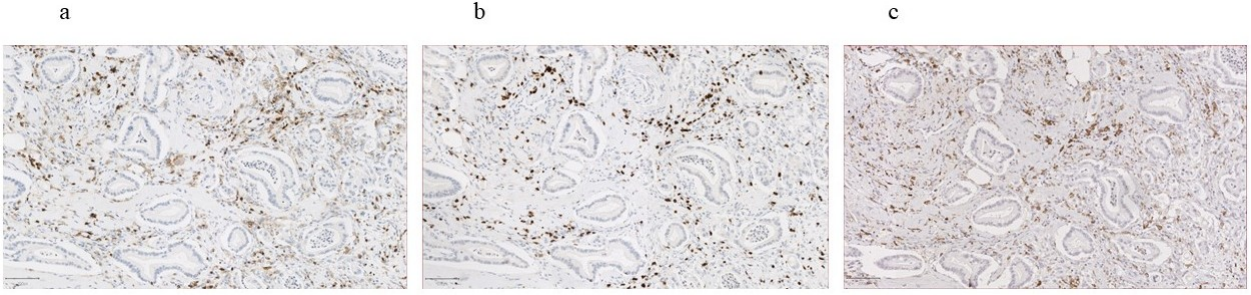
Immune cell populations. a Immunohistochemistry staining for CD4; b Immunohistochemistry staining for CD3; Immunohistochemistry staining for CD163. 20x magnification.

Immunohistochemistry staining of T cell markers CD3, CD4 and macrophage marker CD163 was performed to identify stromal immune cell populations (Fig 6a–6c). The number of CD3 and CD4 positive cells in all stromal areas was evaluated and a ratio of CD4 to CD3 positive cell counts was calculated. CD4/CD3 ratio was dichotomized using the 75 percentile cut-off as previously described [21]. Counts of CD163 positive cells in stromal areas were assessed and the cell counts were dichotomized by the median cell count. Furthermore, immunohistochemistry staining of p53 was performed and the total number of p53 positive cells per slide were calculated. P53 positive cell density was dichotomized according to the 25 percentile. All PDAC specimens were evaluated by all three investigators. In case of disagreement, the dissent was dissolved in discussion.

Overall survival time was defined from surgery until death of any cause.

### Statistics

For statistical analysis, IBM SPSS Statistics for Windows, Version 25.0 was used. Continuous and categorical variables were expressed as median/range and absolute/relative frequencies, respectively. Statistical testing was performed by Chi-squared test or Student’s t-test. Correlation analysis was performed using two-sided Spearman rank test. Median overall survival estimates were determined with Kaplan Meier method. Multivariable analysis was performed with cox proportional hazard model for variables. The significance level was set to p < 0.05 (two-sided). All confidence intervals (CI) reported are 95% confidence intervals.

## Results

### Baseline parameters, histopathology and stroma morphology

A total of 108 patients undergoing oncologic resections for PDAC in the period from 2011 to 2016 were identified, median follow-up was 17.5 months. The median age at operation was 67 years, ranging from 40 to 86. A Whipple procedure was performed in 33 (30.6%) patients, 55 (50.6%) had a pylorus-preserving pancreatoduodenectomy (PPPD), 6 (5.6%) underwent a left pancreatectomy and 14 (13.0%) had a total pancreatectomy.

Median tumor size was 3.0 cm, ranging from 0.8 to 9.0 cm. The most frequent T stage was T2 (54.6%). Most patients showed nodal positive disease (68.4%). 6 (5.6%) patients had distant metastases. Most frequent grading was G2 (54.6%). 23 patients (21.3%) had positive resection margins. 33 (30.6%) patients had parallel alignment of stroma fibers while 79 (69.4%) patients showed random orientation of stroma fibers. In 41 patients (37.3%) stromal alignment was heterogeneous within one slide. In these cases, the slide was classified according to the dominant stromal alignment pattern. Only 7 (6.5%) of 41 patients with heterogeneous stromal alignment had mainly parallel stromal fibers while 34 (31.5%) patients showed mainly random orientation of stromal fibers. To determine the accuracy of stromal alignment assessment in H&E slides, we repeated the analysis of stromal fiber alignment after staining for collagen I. 31.8% of the patients were assessed as showing parallel alignment and 68.2% were determined with random orientation of stroma fibers. Consequently, accuracy of stromal fiber assessment in H&E stained slides is sufficient, and further staining of collagen I does not yield additional benefits. Stromal density was high in 62 (57.4%) patients and low in 46 (42.6%) patients. 82 (75.9%) patients had a low fibroblast density while 26 (24.1%) had a high fibroblast density. Fibroblast morphology was round in 52 (48.1%) patients, 56 (51.9%) showed spindle-like fibroblast morphology. Accurate differentiation of round fibroblasts and immune cells in H&E slides may be difficult. After staining for immune cell populations (CD3, CD4, CD163), assessment of fibroblasts morphology was repeated. The identified pattern of fibroblast morphology was changed for only 2 of 108 cases. Mean microvessel density was 10 microvessels / stroma localization. 57 (52.8%) patients showed a lower microvessel density than 10 microvessel/stroma localization, while 51 (47.2%) had a higher microvessel density. 27 (26.4%) patients had a high CD4/CD3 ratio while 53 (50.0%) had a high density of CD163 positive cells. 77 (71.8%) patients had a high density of p53 positive cells. Details of baseline parameters, histopathology and stroma morphology are displayed in Table 1.

**Table 1:** Baseline parameters, histopathology and stroma morphology.

Baseline Characteristics	
Parameter	*Median (range) or n (%)*
Total n	108
**Age**	67 (40–86)
**Sex**	
female	41 (38.0)
male	67 (62.0)
**Operation**	
Whipple	33 (30.6)
PPPD	55 (50.6)
TPE	14 (13.0)
Left pancreatectomy	6 (5.6)
**Tumor Size** in cm	3.0 (0.8–9.0)
**T stage**	
T1	10 (17.6)
T2	59 (54.6)
T3	19 (17.6)
T4	11 (10.2)
**N stage**	
N0	33 (30.6)
N1	50 (46.3)
N2	25 (23.1)
**M1**	6 (5.6)
**Grading**	
G 1	5 (4.6)
G 2	59 (54.6)
G 3	43 (39.8)
G 4	1 (0.9)
**Resection margin**	
R 0	85 (78.7)
R +	23 (21.3)
**Alignment of stroma fibers**	
parallel	33 (30.6)
random	75 (69.4)
**Stromal density**	
low	62 (57.4)
high	46 (42.6)
**Fibroblast density**	
low	82 (75.9)
high	26 (24.1)
**Fibroblast morphology**	
round morphology	52 (48.1)
spindle-like morphology	56 (51.9)
**Microvessel density**	
low	57 (52.8)
high	51 (47.2)
**Random alignment of stroma fibers and low microvessel density**	40 (37.0)
**CD4/CD3 ratio**	
low	81 (73.6)
high	27 (26.4)
**CD 163 positive cell density**	
<median 5368 cells	53 (50.0)
>median 5368 cells	53 (50.0)
**p53 positive cell density**	
low	31 (28.2)
high	77 (71.8)

PPPD: Pylorus-preserving pancreatoduodenectomy; TPE: Total pancreatectomy.

In patients with pancreatoduodenectomy, 30 patients had parallel alignment and 58 patients showed random orientation of stromal fibers. For patients with distal pancreatectomy (5 versus 1 patients) and total pancreatectomy (12 versus 2 patients) the rate of patients with random orientation of stromal fibers was higher as compared to patients undergoing pancreatoduodenectomy.

High microvessel density was identified in 43 of 88 patients with pancreatoduodenectomy (48.8%). In patients with distal pancreatectomy, the rate of patients with high microvessel density was 2 of 6 (33.3%) while 6 of 14 patients (42.8%) with total pancreatectomy had high microvessel density.

### Stroma morphology and histopathology

Patients with random orientation of stroma fibers showed larger median tumor size (3.62 vs. 2.87, p = 0.037). There was a statistical trend for higher T stage in patients with random orientation of stroma fibers (T3/4 33.3% vs. T3/4 15.2%, p = 0.064). Patients with random orientation of stroma fibers were more likely to be diagnosed with local lymph node metastases (76.0% vs. 54.5%, p = 0.040) and had higher rates of tumor positive resection margins (27.7% vs. 9.1%, p = 0.008). Patients with random orientation of stroma fibers and low microvessel density had higher rates of local lymph node metastases as compared to patients not showing these morphological features (82.5% vs. 61.8%, p = 0.031). Details are depicted in Table 2.

**Table 2:** Stroma morphology and histopathology.

	Parallel Alignment of Stroma Fibers	Random Orientation of Stroma Fibers	
	*n (%)/ mean (SE)*	*n (%)/ mean (SE)*	*p-value*
**Tumor size**	2.87 (0.21)	3.62 (0.16)	**0.037**
**T Stage**			
T1-2	28 (84.8)	50 (66.7)	
T3-4	5 (15.2)	25 (33.3)	**0.064**
**N Stage**			
N0	15 (45.5)	18 (24.0)	
N+	18 (54.5)	57 (76.0)	**0.040**
**M Status**			
M0	31 (93.9)	71 (94.7)	
M1	2 (6.1)	4 (5.3)	1.000
**Grading**			
G1-2	23 (69.7)	41 (54.7)	
G3-4	10 (30.3)	34 (45.3)	0.202
**R Status**			
R0	30 (90.9)	55 (73.3)	
R1	3 (9.1)	20 (27.7)	**0.008**
	**High Stromal Matrix Density**	**Low Stromal Matrix Density**	
**Tumor size**	3.36 (0.17)	3.43 (0.20)	0.806
**T Stage**			
T1-2	46 (74.2)	32 (69.6)	
T3-4	16 (25.8)	14 (30.4)	0.666
**N Stage**			
N0	20 (32.3)	13 (28.3)	
N+	42 (67.7)	33 (71.7)	0.679
**M Status**			
M0	59 (95.2)	43 (93.5)	
M1	3 (4.8)	3 (6.5)	0.698
**Grading**			
G1-2	42 (67.7)	22 (47.8)	
G3-4	20 (32.3)	24 (52.2)	0.048
**R Status**			
R0	39 (62.9)	32 (71.1)	
R1	23 (37.1)	13 (28.9)	0.413
	**High Fibroblast Density**	**Low Fibroblast Density**	
**Tumor size**	3.43 (0.41)	2.36 (0.30)	0.595
**T Stage**			
T1-2	59 (72.0)	19 (73.1)	
T3-4	23 (28.0)	7 (26.9)	0.563
**N Stage**			
N0	21 (25.6)	12 (46.2)	
N+	61 (74.4)	14 (53.8)	0.091
**M Status**			
M0	76 (92.7)	26 (100)	
M1	6 (7.3)	0 (0)	0.332
**Grading**			
G1-2	49 (59.8)	15 (57.7)	
G3-4	33 (40.2)	11 (42.3)	1.000
**R Status**			
R0	53 (64.6)	18 (72.0)	
R1	29 (35.4)	7 (28.0)	0.630
	**Spindle-like Fibroblast Morphology**	**Large Round Fibroblast Morphology**	
**Tumor size**	3.60 (0.14)	3.19 (0.18)	0.122
**T Stage**			
T1-2	39 (75.0)	39 (69.6)	
T3-4	13 (25.0)	17 (30.4)	0.668
**N Stage**			
N0	16 (30.8)	17 (30.4)	
N+	36 (69.2)	39 (69.6)	1.000
**M Status**			
M0	48 (92.3)	54 (96.4)	
M1	4 (7.7)	2 (3.6)	0.425
**Grading**			
G1-2	29 (55.8)	35 (62.5)	
G3-4	23 (44.2)	21 (37.5)	0.558
**R Status**			
R0	36 (69.2)	35 (63.6)	
R1	16 (30.8)	20 (36.4)	0.683
	**Low Microvessel Density**	**High Microvessel Density**	
**Tumor size**	3.30 (0.17)	3.48 (0.20)	
**T Stage**			
T1-2	40 (70.2)	38 (74.5)	
T3-4	17 (28.8)	13 (25.5)	0.671
**N Stage**			
N0	13 (22.8)	20 (39.2)	
N+	44 (77.2)	31 (60.8)	0.094
**M Status**			
M0	55 (96.5)	47 (92.2)	
M1	2 (3.5)	4 (7.8)	0.418
**Grading**			
G1-2	38 (66.7)	26 (51.0)	
G3-4	19 (33.3)	25 (49.0)	0.118
**R Status**			
R0	36 (63.2)	35 (70.0)	
R1	21 (36.8)	15 (30.0)	0.282
	**Random orientation of stroma fibers and low microvessel density**		
	**Absent**	**Present**	
**Tumor size**	3.34 (0.17)	3.47 (0.22)	0.797
**T Stage**			
T1-2	52 (76.5)	26 (65.0)	
T3-4	16 (23.5)	14 (35.0)	0.266
**N Stage**			
N0	26 (38.2)	7 (17.5)	
N+	42 (61.8)	33 (82.5)	**0.031**
**M Status**			
M0	63 (92.6)	39 (97.5)	
M1	5 (7.4)	1 (2.5)	0.409
**Grading**			
G1-2	40 (58.8)	24 (60.0)	
G3-4	28 (41.2)	16 (40.0)	1.000
**R Status**			
R0	48 (70.6)	23 (57.5)	
R1	20 (29.4)	17 (52.5)	0.209

Alignment of stroma fibers was not correlated with CD4/CD3 ratio (CC -0.053, p = 0.593), density of CD163 positive cells (CC -0.15, p = 0.882) or density of p53 positive cells (CC -0.99, p = 0.317).

### Survival analysis

**Fig 7:**
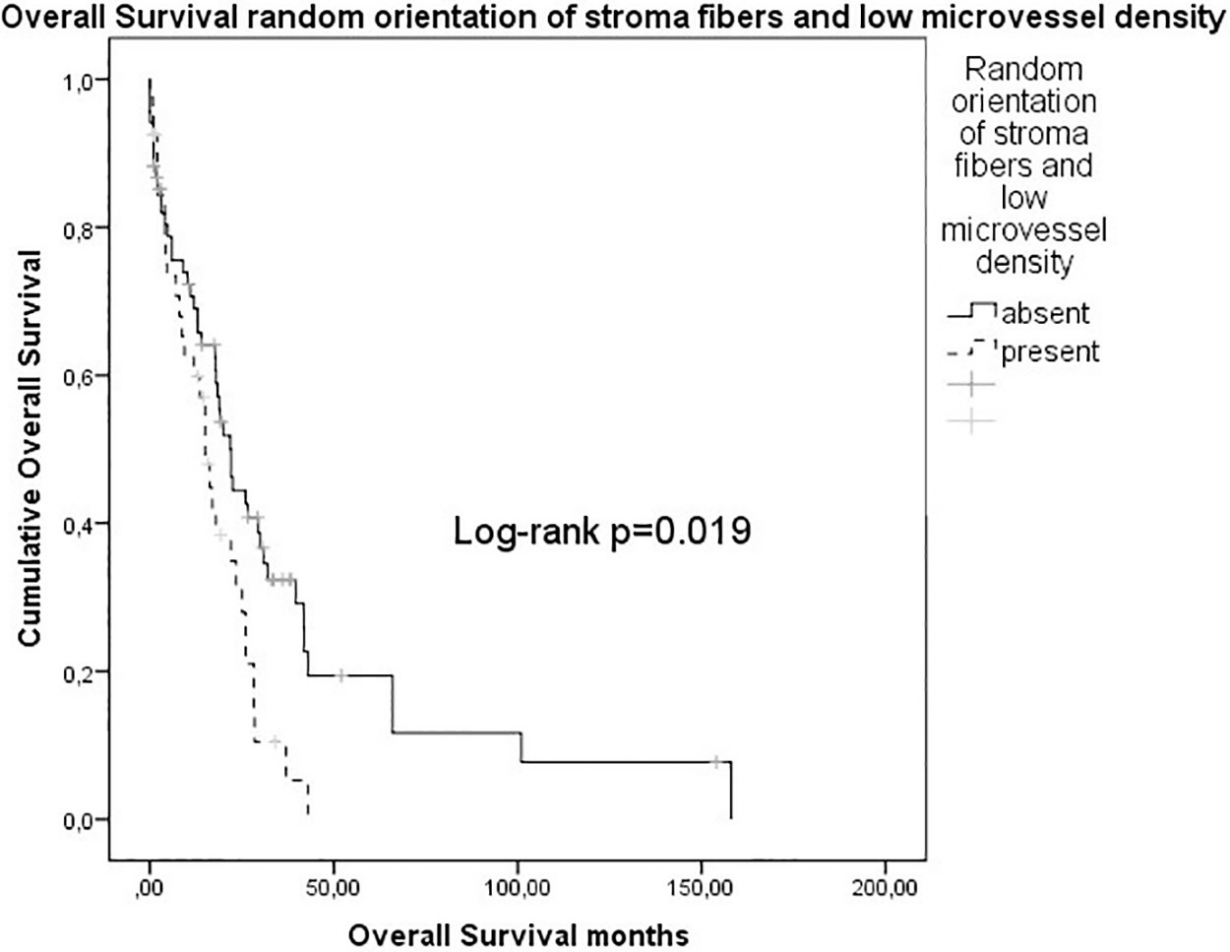
Overall survival random orientation of stroma fibers and low microvessel density.

Median overall survival (OS) of all patients was 15.4 months. Details of survival analysis are depicted in Table 3. N stage (N0 39.1 months vs. N+ 24.1 months, p = 0.022), R status (R0 30.9 months vs. R+ 23.8 months, p = 0.022) and alignment of stroma fibers (parallel alignment 42.0 months vs. random orientation 22.0 months, p = 0.046) qualified as prognostic parameters. There was a statistical trend for improved overall survival in patients with high microvessel density (36.0 months vs. 26.0 months, p = 0.061). Patients with random orientation of stroma fibers and low microvessel density had reduced overall survival rates as compared to patients not showing these stroma features (16.2 months vs. 36.0 months, p = 0.019) (Fig 7). No difference in overall survival was disclosed for patients with heterogeneous versus homogeneous alignment of stromal fibers (28 months versus 33 months, p = 0.167). A high CD4/CD3 ratio was associated with reduced overall survival rates (16 months vs. 33 months, p = 0.040). Patients with high stromal density of CD163 cells had a poor prognosis (27 months vs. 34 months, p = 0.039). In multivariable analysis, the combination of random orientation of stroma fibers and low microvessel density (HR 1.592, 95%CI 1.098–2.733, p = 0.029), high CD4/CD3 ratio (HR 2.044, 95%CI 1.203–3.508, p = 0.028) and high density of CD163 positive cells (HR 1.596, 95%CI 1.367–1.968, p = 0.036) remained independent prognostic factors.

**Table 3:** Survival analysis.

Long-term survival estimates								
	Univariate				Multivariate			
Parameter	*HR*	*95%CI (lower*, *upper)*		*p-value*	*HR*	*95%CI (lower*, *upper)*		*p-value*
**Age**								
< median 67								
> median 67	1.519	0.970	2.831	0.068				
**Sex**								
female								
male	1.608	1.016	2.542	0.142				
**Tumor Size** in cm								
< median 3.0								
> median 3.0	1.348	1.159	2.114	**0.036**	1.225	0.773	1.942	0.388
**T stage**								
T1/2								
T3/4	0.732	0.440	1.217	0.723				
**N stage**								
N0								
N+	1.568	1.260	2.621	**0.022**	1.366	0.791	2.359	0.357
**M**								
M0								
M1	1.024	0.425	3.404	0.727				
**Grading**								
G 1/2								
G 3/4	1.627	1.032	2.532	0.336				
**Resection margin**								
R 0								
R 1	1.343	1.136	2.157	**0.022**	1.215	0.750	1.967	0.430
**Alignment of stroma fibers**								
random								
parallel	1.443	1.280	2.364	**0.046**	1.220	0.719	2.072	0.079
**Stromal density**								
low								
high	1.001	0.633	1.518	0.998				
**Fibroblast density**								
low								
high	1.350	0.817	2.232	0.241				
**Fibroblast morphology**								
round morphology								
spindle-like morphology	1.002	0.643	1.561	0.994				
**Microvessel density**								
low								
high	0.602	0.345	1.050	**0.061**	0.568	0.279	1.157	0.119
**Random orientation of stroma fibers and low microvessel density**								
absent								
present	1.748	1.098	2.785	**0.019**	1.592	1.098	2.733	**0.029**
**CD4/CD3 ratio**								
low								
high	1.686	1.024	2.777	**0.040**	2.044	1.203	3.508	**0.028**
**CD 163 positive cell density**								
low								
high	0.620	0.394	0.975	**0.039**	1.596	1.367	1.968	**0.036**
**p53 positive cell density**								
low								
high	0.974	0.582	1.689	0.919				

## Discussion

The current study systematically evaluated PDAC stroma morphology and defined simple histopathological stroma classification methods. Random orientation of stroma fibers alone results in local tumor progression mirrored by tumor size and higher rates of margin positive resections. A combination of random orientation of stroma fibers and low microvessel density is associated with loco-regional tumor infiltration and qualifies as independent negative prognostic factor. Immune cell infiltrates determine prognosis, and high CD4/CD3 ratio and high counts of CD163 positive tumor-associated macrophages determine prognosis.

In 1986, Dvorak postulated that tumors are "wounds that do not heal" and outlined similarities between carcinogenesis and granulation processes of healing wounds [22]. The author described that in contrast to regular wound healing and scar formation the process in cancer tissue is not self-limiting, but results in tumor progression and invasion. This hypothesis is increasingly supported by current data, in particular for PDAC. Predominant fibrotic areas called desmoplasia are a prominent characteristic of PDAC. In the process of carcinogenesis, local quiescent pancreatic stellate cells are transformed to an active myofibroblast phenotype secreting high amounts of extracellular matrix such as collagens, laminins and fibronectins [6,23]. Such activated cancer-associated fibroblasts and extracellular matrix proteins are major components forming PDAC desmoplasia. Desmoplasia forms a niche for PDAC tumor cells and plays an important role in the promotion of tumor progression and local and systemic tumor cell invasion [7]. The morphological correlate of granulation-mimicking excessive stromal activation in PDAC is a chaotic and disordered stromal architecture with random orientation of stroma fibers. In the current study, this particular morphological stroma feature was identified in a majority of patients (69.4%). Random orientation of stroma fibers correlated with tumor size and lymph node invasion mirroring local progression and loco-regional invasion. Random orientation of stroma fibers was also associated with margin positive resections in PDAC patients. This association is supporting the hypothesis of local tumor progression by excessive desmoplasia that is finally less amenable to complete resection. Fibrotic stromal reaction at the resection margin was recently identified as an obstacle for curative resection [15]. Patients with pronounced fibrosis directly at the resection margin showed an impaired overall survival. In consequence, desmoplasia also plays a role in clinical practice and impairs surgery as the only potentially curative therapy in PDAC.

Morphology of ECM has been increasingly studied over the past years. Stromal alignment studies mainly focused on breast and ovarian cancer [24]. In a mouse model of breast cancer, dense ECM surrounding the tumor characterized early tumor stages while invasive disease showed non-aligned ECM fibers adjacent to the tumor boundary [25]. Several studies on ovarian and breast cancer associated tumor-adjacent parallel stromal alignment with local tumor cell invasion in early stages of tumor progression [24,26–28]. As a limitation, these studies focused on stromal alignment directly at the tumor-stroma boundary and other stromal parts of the cancer specimen were not assessed. Pancreatic cancer stroma is characterized by pronounced stiffness that makes morphological analyses particularly interesting. Stromal stiffness leads to aggressive biological features such as acquisition of a mesenchymal cell type and resistance to chemotherapy in xenograft models of pancreatic cancer [29]. A recent study assessed the impact of stromal fiber orientation on overall survival in PDAC [30]. In contrast to the results of the current study, the authors found an association between parallel alignment of stroma fibers and impaired overall survival. As a limitation, only TMAs, but not entire slides of PDAC specimens were assessed, and main areas of the tumor may have been neglected. Our study is the first to our knowledge to systematically screen entire slides and large stromal areas of cancer specimens. The current study disclosed intra-tumoral heterogeneity in 37.3% of the patients, and a dominant stromal alignment pattern could only be disclosed by assessing different regions of the slide. Furthermore, microvessel morphology was not evaluated in the study. Several studies used a complex multi-step second harmonic generation imaging technique to visualize fibers and to determine their orientation. This stromal fiber evaluation method will be difficult to implement in standard clinical routine. In contrast, the stromal alignment assessment described in the current study is a simple visual classification that can be rapidly integrated to standard pathological work-up.

Patients with parallel alignment of stroma fibers had smaller tumors with less frequent lymph node metastasis and higher rates of R0 resections in the current study. It may be speculated that the desmoplastic transformation during carcinogenesis may have been less pronounced in these patients, resulting in a less tumor promoting stroma. Alignment of stroma fibers qualified as prognostic parameter with a considerable difference in overall survival for patients with random orientation of stroma fibers (22 months) in contrast to patients with parallel alignment of stroma fibers (42 months). However, this stroma feature could not be identified as an independent prognostic parameter. Besides alignment of stroma fibers, also the histopathological variables tumor size, N status and R status all associated with alignment of stroma fibers were prognostic factors in univariate analysis. So, as the most probable cause, these three variables were ruled out in multivariate analysis.

Stroma density was not associated with tumor progression or overall survival. We recently demonstrated that dense stroma limited PDAC tumor cells growth and induced markers of epithelial-to-mesenchymal transition in an *in vitro* model [5]. The only study evaluating the prognostic effect of stromal density on PDAC prognosis was recently performed by Torphy et al. [31]. The authors observed improved progression-free and overall survival rates in patients with dense stroma not undergoing adjuvant therapy. In patients undergoing adjuvant therapy, the survival benefit diminished over time.

Fibroblast density and morphology were not associated with prognosis in the current study. In *in vitro* models transformation from round quiescent fibroblasts to activated spindle-like shaped myofibroblasts as well as fibroblast proliferation can be achieved by co-cultivation with PDAC tumor cells [4,5]. It may be speculated that these processes cannot be verified in mature PDAC tumors but are present in early carcinogenesis. In addition to activated and quiescent fibroblasts, recent studies have identified multiple different subtypes of PDAC-associated fibroblasts bearing tumor-promoting as well as tumor-restraining properties [8,32,33]. These subtypes can probably not be identified by morphological analysis alone.

Hypoxia is an important feature of PDAC, and overall survival decreases by the extent of hypoxic regions in PDAC tumors. In contrast to other solid malignancies, hypoxia is in particular pronounced in PDAC [3,34]. PDAC tumor cells adopt to limited oxygen supply and develop an invasive and chemo-resistant phenotype [35,36]. Desmoplasia contributes to PDAC hypoxia by displacing capillaries with high amounts of extracellular matrix and by hypoxia-associated activation of fibroblasts [35,37,38]. Desmoplasia and hypoxia together form a self-sustaining interaction network supporting PDAC invasiveness and therapeutic resistance. This mechanism is supported by the current study, random orientation of stroma fibers and low microvessel density were associated with lymph node metastasis. Furthermore, the combination of random orientation of stroma fibers and low microvessel density were found to be an independent negative prognostic parameter in this study. In consequence, the combination of pronounced desmoplasia and hypoxia seems to be a key histological aspect of the PDAC microenvironment linked with poor prognosis.

Tumor-infiltrating lymphocytes are part of the PDAC microenvironment, and were shown to impact tumor progression and even patient prognosis [39,40]. CD3 is a co-T-cell-receptor required for activation of both cytotoxic and helper T cells. Recently, a correlation between high CD3 cell counts and favorable pathological characteristics was demonstrated [21]. A recent meta-analysis emphasized the importance of lymphocyte ratios and recommended to evaluate CD3 positive infiltrates in relation to all CD4 positive T helper cells [40]. Our study demonstrated poor prognosis in patients with high CD4/CD3 ratios. These results are consistent with a current study showing reduced disease-free and overall survival in patients with high CD4/CD3 ratios [21].

Tumor-associated macrophages (TAM) form a subset population of the PDAC immune-microenvironment. These cells are characterized by positive CD163 expression and orchestrate tumor evasion from immune surveillance by anti-inflammatory cytokine signaling [41]. In our study, high stromal infiltrates of CD163 positive cells were associated with reduced overall survival. These findings confirm the results of former studies demonstrating poor outcome in PDAC patients with high CD163 cell counts [42,43].

In summary, we present a simple morphological classification method for PDAC stroma. Alignment of stroma fibers and microvessel density were associated with tumor progression, locoregional tumor invasion and surgical resectability. Both random orientation of stroma fibers and low microvessel density qualified as independent prognostic parameters. In consequence, the combination of excessive desmoplasia and hypoxia seems to be of major relevance for PDAC patient prognosis. High CD4/CD3 stromal ratios and high infiltrates of tumor-associated macrophages were associated with reduced overall survival. Simple systematic assessment of stroma morphology may help to assess patient prognosis.

## References

[1] Howlader N, Noone A, Krapcho M, Garshell J, Miller D, Altekruse S. SEER cancer statistics review, 1975–2012, National Cancer Institute. Bethesda. 2015;

[2] Lillemoe KD, Yeo CJ, Cameron JL. Pancreatic cancer: state-of-the-art care. CA Cancer J Clin. 2000;50(4):241–68. 10.3322/canjclin.50.4.241 10986966

[3] Elaileh A, Saharia A, Potter L, Baio F, Ghafel A, Abdelrahim M, et al Promising new treatments for pancreatic cancer in the era of targeted and immune therapies. Am J Cancer Res. 2019;9(9):1871–88. 31598392 PMC6780661

[4] Apte M, Park S, Phillips P, Santucci N, Goldstein D, Kumar R, et al Desmoplastic reaction in pancreatic cancer: role of pancreatic stellate cells. Pancreas. 2004;29(3):179–87. 10.1097/00006676-200410000-00002 15367883

[5] Bolm L, Cigolla S, Wittel UA, Hopt UT, Keck T, Rades D, et al The Role of Fibroblasts in Pancreatic Cancer: Extracellular Matrix Versus Paracrine Factors. Transl Oncol. August 2017;10(4):578–88. 10.1016/j.tranon.2017.04.009 PMC5487255 28658650

[6] Apte M, Xu Z, Pothula S, Goldstein D, Pirola R, Wilson J. Pancreatic cancer: the microenvironment needs attention too! Pancreatology. 2015;15(4):32–8. 10.1016/j.pan.2015.02.013 25845856

[7] Whittle MC, Hingorani SR. Fibroblasts in Pancreatic Ductal Adenocarcinoma: Biological Mechanisms and Therapeutic Targets. Gastroenterology. Mai 2019;156(7):2085–96. 10.1053/j.gastro.2018.12.044 PMC6486863 30721663

[8] Moffitt RA, Marayati R, Flate EL, Volmar KE, Loeza SGH, Hoadley KA, et al Virtual microdissection identifies distinct tumor-and stroma-specific subtypes of pancreatic ductal adenocarcinoma. Nat Genet. 2015;47(10):1168–78. 10.1038/ng.3398 26343385 PMC4912058

[9] Vennin C, Melenec P, Rouet R, Nobis M, Cazet AS, Murphy KJ, et al CAF hierarchy driven by pancreatic cancer cell p53-status creates a pro-metastatic and chemoresistant environment via perlecan. Nat Commun. 2019;10(1):3637 10.1038/s41467-019-10968-6 31406163 PMC6691013

[10] Feig C, Gopinathan A, Neesse A, Chan DS, Cook N, Tuveson DA. The pancreas cancer microenvironment. Clin Cancer Res Off J Am Assoc Cancer Res. 2012;18(16):4266–76. 10.1158/1078-0432.CCR-11-3114 PMC3442232 22896693

[11] Xing F, Saidou J, Watabe K. Cancer associated fibroblasts (CAFs) in tumor microenvironment. Front Biosci J Virtual Libr. 2010;15:166–79. 10.2741/3613 PMC2905156 20036813

[12] Castelló-Cros R, Cukierman E. Stromagenesis during tumorigenesis: characterization of tumor-associated fibroblasts and stroma-derived 3D matrices. Methods Mol Biol. 2009; 522:275–305. 10.1007/978-1-59745-413-1_19 19247611 PMC2670062

[13] Alifano M, Mansuet-Lupo A, Lococo F, Roche N, Bobbio A, Canny E, et al Systemic inflammation, nutritional status and tumor immune microenvironment determine outcome of resected non-small cell lung cancer. PloS One. 2014;9(9). 10.1371/journal.pone.0106914 PMC4169516 25238252

[14] Clark CE, Hingorani SR, Mick R, Combs C, Tuveson DA, Vonderheide RH. Dynamics of the immune reaction to pancreatic cancer from inception to invasion. Cancer Res. 2007;67(19):9518–27. 10.1158/0008-5472.CAN-07-0175 17909062

[15] Wellner UF, Krauss T, Csanadi A, Lapshyn H, Bolm L, Timme S, et al Mesopancreatic stromal clearance defines curative resection of pancreatic head cancer and can be predicted preoperatively by radiologic parameters: a retrospective study. Medicine (Baltimore). 2016;95(3). 10.1097/MD.0000000000002529 PMC4998270 26817896

[16] Moffitt RA, Marayati R, Flate EL, Volmar KE, Loeza SGH, Hoadley KA, et al Virtual microdissection identifies distinct tumor-and stroma-specific subtypes of pancreatic ductal adenocarcinoma. Nat Genet. 2015;47(10):1168–78. 10.1038/ng.3398 26343385 PMC4912058

[17] Sr Broders A. Malignant neoplasia of normally situated and heterotopic lymphoid tissue and its numerical microscopic grading. Tex State J Med. 1953;49(4):234 13049202

[18] Edge SB, Compton CC. The American Joint Committee on Cancer: the 7th edition of the AJCC cancer staging manual and the future of TNM. Ann Surg Oncol. 2010;17(6):1471–4. 10.1245/s10434-010-0985-4 20180029

[19] Bankhead P, Loughrey MB, Fernandez JA, Dombrowski Y, McArt DG, Dunne PD, et al QuPath: Open source software for digital pathology image analysis. Sci Rep. 2017;7(1):16878 10.1038/s41598-017-17204-5 29203879 PMC5715110

[20] Weidner N. Intratumor microvessel density as a prognostic factor in cancer. Am J Pathol. 1995;147(1):9–19. 7541613 PMC1869874

[21] Delayre T, Guilbaud T, Resseguier N, Mamessier E, Rubis M, Moutardier V, et al Prognostic impact of tumour-infiltrating lymphocytes and cancer-associated fibroblasts in patients with pancreatic adenocarcinoma of the body and tail undergoing resection. Br J Surg. 2020 5;107(6):720–733. 10.1002/bjs.11434 31960955

[22] Dvorak HF. Tumors: wounds that do not heal. Similarities between tumor stroma generation and wound healing. N Engl J Med. 1986;315(26):1650–9. 3537791 10.1056/NEJM198612253152606

[23] Melstrom LG, Salazar MD, Diamond DJ. The pancreatic cancer microenvironment: A true double agent. J Surg Oncol. 2017;116(1):7–15. 10.1002/jso.24643 28605029 PMC5989710

[24] Tilbury K, Campagnola PJ. Applications of second-harmonic generation imaging microscopy in ovarian and breast cancer. Perspect Med Chem. 2015;7:21–32. 10.4137/PMC.S13214 PMC4403703 25987830

[25] Provenzano PP, Eliceiri KW, Campbell JM, Inman DR, White JG, Keely PJ. Collagen reorganization at the tumor-stromal interface facilitates local invasion. BMC Med. 2006;4(1):38 10.1186/1741-7015-4-38 17190588 PMC1781458

[26] Conklin MW, Eickhoff JC, Riching KM, Pehlke CA, Eliceiri KW, Provenzano PP, et al Aligned collagen is a prognostic signature for survival in human breast carcinoma. Am J Pathol. 2011;178(3):1221–32. 10.1016/j.ajpath.2010.11.076 21356373 PMC3070581

[27] Bredfeldt JS, Liu Y, Conklin MW, Keely PJ, Mackie TR, Eliceiri KW. Automated quantification of aligned collagen for human breast carcinoma prognosis. J Pathol Inform. 2014;5(1):28 10.4103/2153-3539.139707 25250186 PMC4168643

[28] Burke K, Tang P, Brown E. Second harmonic generation reveals matrix alterations during breast tumor progression. J Biomed Opt. 2013;18(3):31106 10.1117/1.JBO.18.3.031106 23172133 PMC3595714

[29] Rice AJ, Cortes E, Lachowski D, Cheung BCH, Karim SA, Morton JP, et al Matrix stiffness induces epithelial-mesenchymal transition and promotes chemoresistance in pancreatic cancer cells. Oncogenesis. 2017;6(7):e352 10.1038/oncsis.2017.54 28671675 PMC5541706

[30] Drifka CR, Loeffler AG, Mathewson K, Keikhosravi A, Eickhoff JC, Liu Y, et al Highly aligned stromal collagen is a negative prognostic factor following pancreatic ductal adenocarcinoma resection. Oncotarget. 2016;7(46):76197–213. 10.18632/oncotarget.12772 27776346 PMC5342807

[31] Torphy RJ, Wang Z, True-Yasaki A, Volmar KE, Rashid N, Yeh B, et al Stromal Content Is Correlated With Tissue Site, Contrast Retention, and Survival in Pancreatic Adenocarcinoma. JCO Precis Oncol. 2018;10:e1200. 10.1200/PO.17.00121 PMC6262879 30506016

[32] Collisson EA, Sadanandam A, Olson P, Gibb WJ, Truitt M, Gu S, et al Subtypes of pancreatic ductal adenocarcinoma and their differing responses to therapy. Nat Med. 2011;17(4):500–3. 10.1038/nm.2344 21460848 PMC3755490

[33] Ligorio M, Sil S, Malagon-Lopez J, Nieman LT, Misale S, Di Pilato M, et al Stromal Microenvironment Shapes the Intratumoral Architecture of Pancreatic Cancer. Cell. 2019;178(1):160–175.e27. 10.1016/j.cell.2019.05.012 31155233 PMC6697165

[34] Hirakawa T, Yashiro M, Doi Y, Kinoshita H, Morisaki T, Fukuoka T, et al Pancreatic Fibroblasts Stimulate the Motility of Pancreatic Cancer Cells through IGF1/IGF1R Signaling under Hypoxia. PloS One. 2016;11(8):e0159912. 27487118 10.1371/journal.pone.0159912 PMC4972430

[35] Erkan M, Kurtoglu M, Kleeff J. The role of hypoxia in pancreatic cancer: a potential therapeutic target? Expert Rev Gastroenterol Hepatol. 2016;10(3):301–16. 10.1586/17474124.2016.1117386 26560854

[36] Erickson LA, Highsmith WEJ, Fei P, Zhang J. Targeting the hypoxia pathway to treat pancreatic cancer. Drug Des Devel Ther. 2015;9:2029–31. 10.2147/DDDT.S80888 25897209 PMC4396576

[37] Rhim AD, Oberstein PE, Thomas DH, Mirek ET, Palermo CF, Sastra SA, et al Stromal elements act to restrain, rather than support, pancreatic ductal adenocarcinoma. Cancer Cell. 2014;25(6):735–47. 10.1016/j.ccr.2014.04.021 24856585 PMC4096698

[38] Baril P, Gangeswaran R, Mahon P, Caulee K, Kocher H, Harada T, et al Periostin promotes invasiveness and resistance of pancreatic cancer cells to hypoxia-induced cell death: role of the β 4 integrin and the PI3k pathway. Oncogene. 2007;26(14):2082–94. 10.1038/sj.onc.1210009 17043657

[39] Hwang HK, Kim H-I, Kim SH, Choi J, Kang CM, Kim KS, et al Prognostic impact of the tumor-infiltrating regulatory T-cell (Foxp3(+))/activated cytotoxic T lymphocyte (granzyme B(+)) ratio on resected left-sided pancreatic cancer. Oncol Lett. 2016;12(6):4477–84. 10.3892/ol.2016.5252 28105157 PMC5228542

[40] Gooden MJM, de Bock GH, Leffers N, Daemen T, Nijman HW. The prognostic influence of tumour-infiltrating lymphocytes in cancer: a systematic review with meta-analysis. Br J Cancer. 2011;105(1):93–103. 10.1038/bjc.2011.189 21629244 PMC3137407

[41] Tjomsland V, Niklasson L, Sandstrom P, Borch K, Druid H, Bratthall C, et al The desmoplastic stroma plays an essential role in the accumulation and modulation of infiltrated immune cells in pancreatic adenocarcinoma. Clin Dev Immunol. 2011;e212810. 10.1155/2011/212810 PMC3235447 22190968

[42] Jamieson NB, Mohamed M, Oien KA, Foulis AK, Dickson EJ, Imrie CW, et al The relationship between tumor inflammatory cell infiltrate and outcome in patients with pancreatic ductal adenocarcinoma. Ann Surg Oncol. 2012;19(11):3581–90. 10.1245/s10434-012-2370-y 22555345

[43] Xu J-Y, Wang W-S, Zhou J, Liu C-Y, Shi J-L, Lu P-H, et al The Importance of a Conjoint Analysis of Tumor-Associated Macrophages and Immune Checkpoints in Pancreatic Cancer. Pancreas. 2019;48(7):904–12. 10.1097/MPA.0000000000001364 31268976

